# Supplementary material for: Tectoridin Stimulates the Activity of Human Dermal Papilla Cells and Promotes Hair Shaft Elongation in Mouse Vibrissae Hair Follicle Culture
Source: Molecules. 2022 Jan 8;27(2):400. doi: 10.3390/molecules27020400 (PMC8778330; doi:10.3390/molecules27020400)
Supplement: Supplementary file 1 [file molecules-27-00400-s001.zip › molecules-1491975-supplementary.pdf]

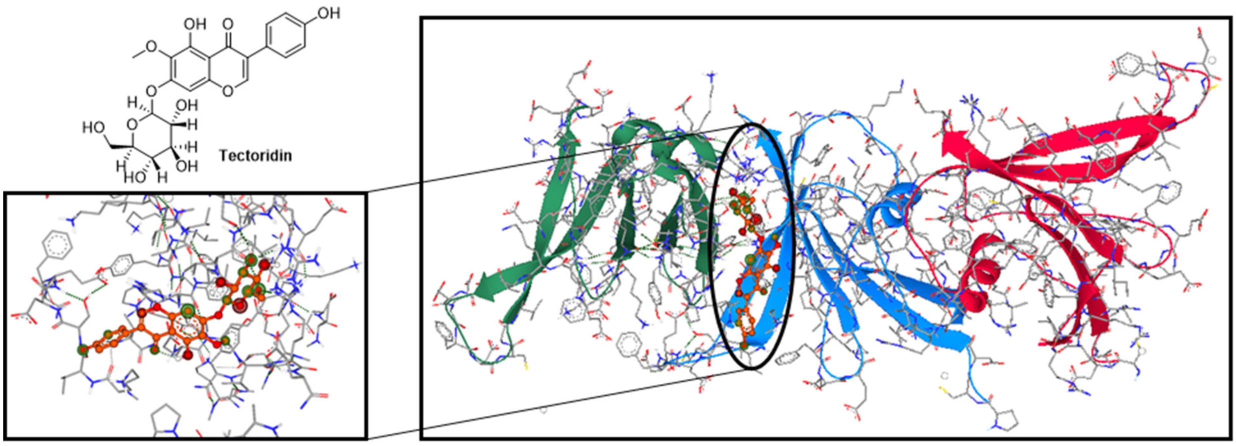

Supplementary Figure S1. Docking image of tectoridin against AXIN2 protein.

SEESAE was employed as a main docking software, and the structure of AXIN2 protein was downloaded from PDB (code: 1WSP); while the structure of tectoridin was generated from Chemdraw. Phytochemicals were subjected to molecular docking analysis. Tectoridin was predicted as the lowest estimated free energy for the binding (- 12.3 KJ/mol), following by resveratrol (-11.6 KJ/mol), mangiferin (-11.5 KJ/mol), tectorigenin (-9 KJ/mol), irigenin (-8.6 KJ/mol), 7-O-methylmangiferin (weak binding) and irisflorentin (weak binding).

Figure S1

Yuen et al 2021

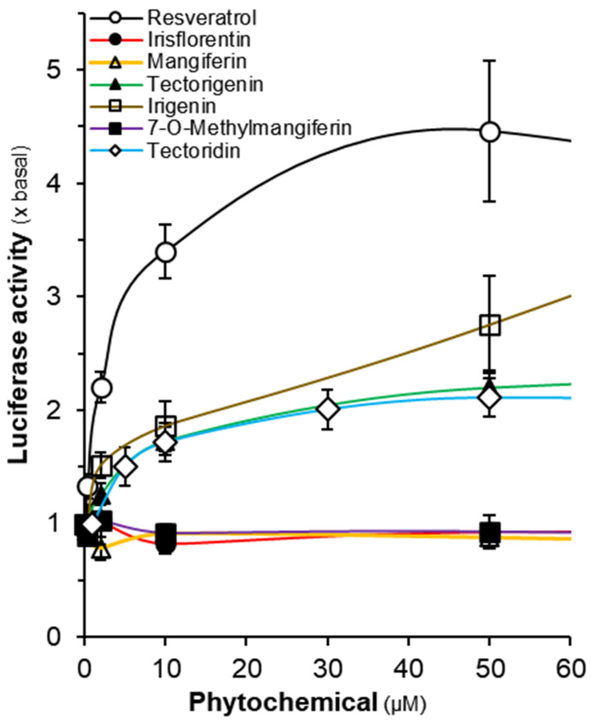

Supplementary Figure S2. Luciferase reporter assay of the phytochemicals in Rhizoma Belamcandae

HEK293T cells were transfected with pTOPFLASH DNA construct for 4 hours. The cultures were treated with different phytochemicals, identified in Rhizoma Belamcandae, for another 24 hours. The cell lysate was subjected for luciferase assays. Data are normalized and expressed as the fold (x basal) of control (control group was treated with 0.02% DMSO), in mean ± SEM, *n* = 4, each with triplicate samples.

Figure S2

Yuen et al 2021

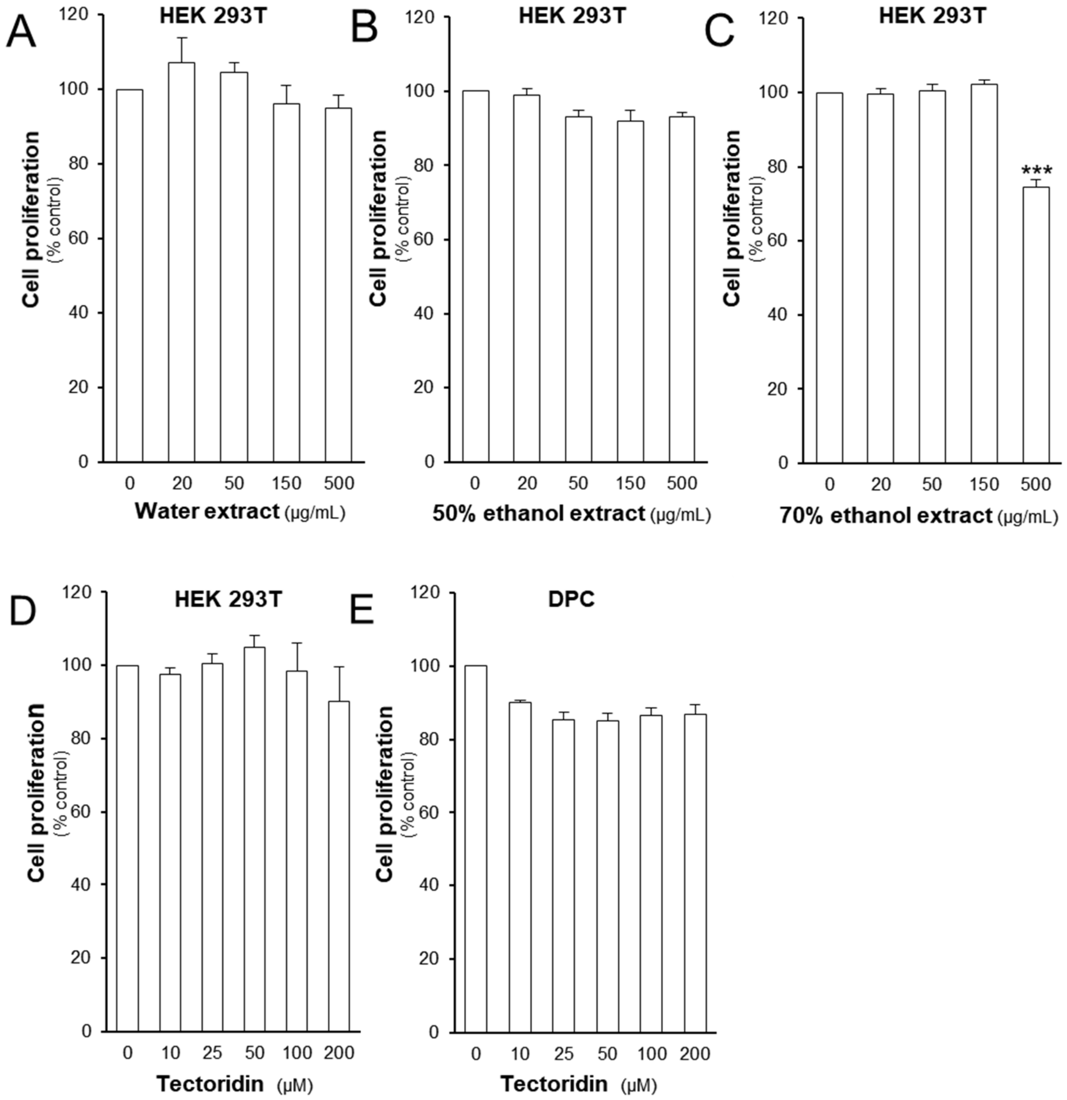

Supplementary Figure S3. Cytotoxicity of extracts of Rhizoma Belamcandae and tectoridin in cultures.
Different concentrations of the extracts of Rhizoma Belamcandae, or tectoridin, were applied onto cultured DPC or HEK293T cells for 24 hours. MTT assay was performed. Data are normalized and expressed as the % of control (control group was treated with 0.02% DMSO), in mean ± SEM, *n* = 4, each with triplicate samples. ****p* < 0.001.

Figure S3

Yuen et al 2021
